# Musical rhythms and their influence on P300 velocity in young females

**DOI:** 10.1590/S1808-86942011000200004

**Published:** 2015-10-19

**Authors:** Cintia Ishii de Sá, Liliane Desguado Pereira

**Affiliations:** 1 Master's degree in Sciences, graduate program on Human Communications Disorders, Speech therapist; 2 Associate professor, Hearing Disorders Discipline, Sao Paulo Federal University. Sao Paulo Federal University (Universidade Federal de São Paulo)

**Keywords:** evoked potentials, auditory, event-related potentials, p300, hearing

## Abstract

Exposure to music may be useful in the P300 retest and avoid habituation.

**Aim:**

To verify the influence of the exposure to different kinds of music in P300 in young females.

**Study design:**

Clinical prospective.

**Material and Method:**

Forty-five women aged from 20 to 36 years were evaluated. P300 was studied before and after musical stimulation with different rhythms. Brazilian songs, international songs, and classical music melodies were selected. Each song had its velocity altered and was named as fast and slow. Subjects were divided into 2 groups exposed to music: one group was exposed to the fast version and the other to the slow version. The control group not exposed to music and was evaluated within the same time period of the others.

**Result:**

There were statistically significant differences when comparing P300 amplitude in the first and third stimulation with the comparison group.

**Conclusion:**

In the same subject, several sequential registrations of P300 caused habituation, which was not seen during exposure to music before P300 recording. Exposure to music at preset different velocities did not affect the P300 in young females.

## INTRODUCTION

There are studies on the effect of music on our body, as exposure to music activates brain areas related with attention, semantics, music analysis, memory, and motor functions.^1^ It also benefits multimode stimulation (hearing, vision, and olfaction) for cognitive and motor rehabilitation,^2^ and helps prevent anxiety, depression, and pain.^3^

Musical rhythms alter noradrenalin levels, one of the neurotransmitters involved with the state of alertness^4^ and memory formation.^5^ Estrogen concentration also affects noradrenalin by modifying female behaviors such as mood and cognition.^6^

The P300 wave is a long latency auditory evoked potential that is generated by discriminating a rare auditory stimulus among frequent auditory stimuli.^7^, ^8^ It also yields information about the auditory system, and has been a tool for investigating selective attention, information processing, and cognition.^9^ Hormone variations, such as estrogen concentrations, may affect the P300 wave.^10^

As noradrenalin and estrogen concentrations modulate brain activity and are part of the behavioral and physiological responses of the central nervous system, and as musical rhythms may affect attention and memory, there is an interest in studying whether they affect the P300 wave cognitive potential as a response to central nervous system function. Thus, the purpose of this study was to verify the influence of exposure to different types of music at varying speeds on P300 wave measurements in young female subjects.

## MATERIALS AND METHODS

The institutional review board approved this study (no. 0743/07), which abided by the ethical principles for research in human beings.

The tests were done at the Human Hearing Disorders Outpatient Unit (Ambulatório de Distúrbios da Audição Humana). The series comprised 45 female subjects aged from 20 to 36 years, of middle to full higher education. The inclusion criteria were: normal hearing on the basic audiological evaluation (pure tone audiometry, logoaudiometry, and acoustic immittance testing); no changes in central auditory function (digit dichotic test, duration pattern, and the random gap detection test); no complaints associated with voice, language, speech or hearing.

Testing was done during the time of low estrogen and progesterone levels in women not using oral contraceptives (early follicular subphase - from the beginning of menstruation to the 5^th^ day; and late luteal subphase - up to 4 days before the beginning of menstruation) to avoid changes because of hormone variations in the ovarian cycle. Testing was done at any time in women taking oral contraceptives, which keep hormone levels stables throughout the cycle.^11^

A four-channel Model 92 Biologic Systems Corp. device with the Evoked Potential System version 5.70 software was used to evaluate P300.

Recording of the P300 wave was done by placing the electrodes in the positions Fz (ground electrode), Cz (positive electrode), and A2 and A1 (negative electrodes) based on the 10-20 international standard,^12^ with a maximum impedance of 5 kohms for each electrode, and a 3 kohms difference between electrodes. A jumper was used in the recording channel of the positive electrode (input 1). Tone bursts were presented through ER-3A 70 dB HL insert earphones. The number of sweeps was 300 stimuli, the frequent stimulus frequency was 1000 Hz, and the rare stimulus frequency was 2000 Hz, presented in an oddball paradigm with an 80% and 20% probability. Participants were asked to remain awake with closed eyes in a silent room and to mentally count the rare stimuli. As the sample consisted of normal hearing subjects, and the derivations (Cz/A1 and Cz/A2) were similar, we chose to add the resulting derivations to obtain a value in milliseconds (ms) for the P300 wave latency (P300 - LAT) and a value in microvolts (µV) for the P300 wave amplitude (P300 - AMP).

We chose three types of music to which subjects were exposed: Brazilian music (BM), non-Brazilian music (NBM), and classical music (CM). Using the Virtual DJ software, we identified the beat per minute (bpm) for each song; we decreased the bpm for slow music and increased the bpm for rapid songs (Frame 1).

**Frame 1:** Speeds of different types of music.

Music	Original bpm	Slow bpm	Rapid bpm
Brazilian music	140.1	130.1	150.1
Non-Brazilian music	167.3	157.3	177.3
Classical music	177.3	167.3	187.3

A Phillips EXP 322/model 19 mp3 CD player with earphones, at volume 5, was used for presenting the songs.

The series was divided into three groups of 15 subjects each: one exposed to three types of music at slower speeds (SMG), one exposed to the same songs at a higher speed (FMG), and a group not exposed to music (NMG - comparison group).

There were four moments during testing. To begin with, SMG and FMG participants were at rest (REP) during which the P300 wave was recorded. The first song was presented during 10 minutes and the P300 wave was rerecorded (second moment, or the first response following the first music stimulus). The third moment consisted of a 5-minute interval followed by the second song lasting 10 minutes. The P300 wave was rerecorded (second response after the second music stimulus). The fourth moment was another 5-minute interval followed by the third song presented during 10 minutes. The P300 wave was recorded again (third response after the third music stimulus).

We controlled the sequence of stimuli so that the song presentation order did not affect the results. The music sequence for the first participant was BM, NBM, and CC. The music sequence for the second participant was CM, BM, and NBM. The music sequence for the third participant was NBM, CM, and BM. This approach continued until the last participant.

For the NMG we recorded the P300 wave without a music stimulus. The interval times between songs was also followed (totaling 15 minutes) to check whether these intervals altered the P300 wave.

The statistical analysis was done using analysis of variance (ANOVA), a parametric test to compare means using variance. The ANOVA one-way parametric test was used for comparing the variance of means among groups. The descriptive analysis of data was done with 95% confidence intervals and a 5% significance level. The Bonferroni correction was applied in multiple comparisons.

## RESULTS

No statistically significant differences were found in comparisons of the LAT-P300 at all testing moments in each group (ANOVA) (*p*-values: FMG = 0.595; SMG= 0.402; NMG = 0.771).

There was a statistically significant difference in the NMG, but no statistically significant difference in FMG and SMG, in the comparison of AMP-P300 at different testing moments (Table 1) (*p*-values: FMG = 0.355; SMG = 0.238).

**Table 1:** *p*-value amplitude at testing moments in the NMG.

NMG		Mean	Median	Standard deviation	VC	Min	Max	CI	*p*-value
P300 - AMP em *μ*V	Rep	17.34	16.61	4.27	24.6%	10.39	24.9	2.16	0,011*
	1^a^	15.78	16.08	4.10	26.0%	9.07	22.97	2.08	
	2^a^	14.85	14.55	5.45	36.7%	7.21	27	2.76	
	3^a^	13.40	13.54	5.14	38.4%	5.09	26.98	2.60	

* significant *p*-value (ANOVA test)

Bonferroni's multiple comparisons were applied (Table 2), the difference occurs between rest and after the third music stimulus.

**Table 2:** Comparison of p-values at testing moments for the amplitude in the NMG.

NMG		Rep	1^a^	2^a^
P300 - AMP	1^a^	1.000		
	2^a^	0.416	1.000	
	3^a^	0.019*	0.187	0.125

* significant *p*-value was - comparisons of the Bonferroni test

A comparison between the speed of songs and the derivations showed that LAT-P300 and AMP-P300 in the SMG trended towards significance according to the ANOVA test (Table 3).

**Table 3:** *p*-values for the variables LAT-P300 and AMP-P300 for SMG.

SMG		Mean	Median	Standard deviation	VC	Min	Max	CI	*p*-value
LAT - P300 in ms	Rep	293.80	289.2	31.34	10.7%	232.2	350.2	15.86	0,063#
	BM	302.67	308.2	37.48	12.4%	224.2	345.2	18.97	
	NBM	304.27	309.2	34.09	11.2%	238.2	351.2	17.25	
	CM	308.93	318.2	32.09	10.4%	245.2	347.2	16.24	
AMP - P300 in *μ*V	Rep	15.62	15	6.68	42.7%	4.27	26.31	3.38	0,066#
	BM	13.07	12.61	4.29	32.8%	5.74	18.94	2.17	
	NBM	12.96	12.83	4.44	34.3%	4.89	21.56	2.25	
	CM	14.91	15.16	5.42	36.3%	5.8	26.87	2.74	

# *p*-value trending towards significance in the ANOVA test.

There was no significance or trend towards significance in a comparison between music speed and recorded derivations in the FMG (*p*-values: LAT-P300 = 0.874 and AMP-P300 = 0.262).

The ANOVA one-way test showed no statistically significant difference when comparing the different testing moments (rest, 1^st^, 2n, and 3^rd^) among the three groups (NMG, FMG, and SMG) for the variables P300-LAT (*p*-values: rest=0.795, 1^st^=0.945, 2^nd^=0.453, 3^rd^=0.859) and P300-AMP (*p*-values: rest=0.295, 1^st^=0.140, 2^nd^=0.319, 3^rd^=0.671). A comparison among groups revealed similar performances for each electrophysiological measure at all testing moments.

The ANOVA one-way test comparing the type of music (BM, NBM, and CM) and music speeds (FMG and SMG) showed no statistically significant differences in P300-LAT (*p*-values: rest=0.338, BM=0.787, NBM=0.537, CM=0.486) and P300-AMP (*p*-values: rest=0.494, BM=0.321, NBM=0.877, CM=0.143). Thus, a comparison of different music speeds and types showed similar electrophysiological measurements in groups FMG and SMG.

## DISCUSSION

Recording the P300 wave after several stimuli may alter neuron network function and result in mental fatigue, which affects the P300 wave amplitude; this is the habituation phenomenon.^13^ We assume that the NMG had habituation, as no music stimuli were given between recordings, resulting in decreased attention for the task.

Even with habituation, reproduction of the P300 wave after several measurements showing latency and amplitude value differences suggests that the P300 wave is a reliable auditory evoked potential, since recorded responses may be tested and confirmed.^14^

In most published studies,^15^, ^16^, ^17^, ^18^ music is presented at the same time as auditory evoked potentials are recorded; music is therefore more of an auditory distraction, rather than a prior condition for recordings. Therefore, we may infer that music stimulation before recording the P300 wave is not so much a distracting factor, but a method for directing the subject's attention to a new P300 task - which did not happen in the NMG.

A study of the effects of acoustic stimulation following noise-induced hearing loss in cats with the introduction of an acoustic spectrum corresponding to the hearing loss frequency showed that auditory thresholds improved, with a normal tonotopic map; it was concluded that such stimulation prevented tinnitus.^16^ We may also infer that exposure to music before recording the P300 wave fosters neural synchronism and stimulates the tonotopic map of frequencies, thereby facilitating the test.

The statistical analysis showed a trend towards significance in the SMG - increased latency and decreased amplitude. This trend suggests that rapid speeds are more beneficial for attention. A study on the effect of different types of music on the P300 wave^15^ showed that exposure to familiar music resulted in a positive effect over the cognitive potential, favoring selective attention and memory processes.

Based on our findings, we suggest that when the P300 wave derivation is difficult to find, examiners may use music stimulation before reevaluating this wave. When music was not available between recordings, the P300 wave was recorded with a higher latency and lower amplitude.

Given that this study included only young women, we suggest additional studies of the P300 wave in subjects with neurological conditions.

## CONCLUSION

Exposure to music at varying preestablished speeds did not alter P300 in young female adults. A comparison of performance among groups showed that exposure to music before measuring P300 facilitated attention and sustained attention in testing, which did not occur in women that were not exposed to music before the test, as there was habituation to P300. Thus, exposure to music could facilitate a reevaluation of the P300 wave.
